# Anti-Fibrotic and Anti-Inflammatory Effects of Hesperidin in an Ex Vivo Mouse Model of Early-Onset Liver Fibrosis

**DOI:** 10.3390/ijms27020594

**Published:** 2026-01-07

**Authors:** Ilenia Saponara, Miriam Cofano, Valentina De Nunzio, Giusy Bianco, Raffaele Armentano, Giuliano Pinto, Emanuela Aloisio Caruso, Matteo Centonze, Maria Notarnicola

**Affiliations:** 1 Laboratory of Nutritional Biochemistry, National Institute of Gastroenterology IRCCS “Saverio de Bellis”, 70013 Castellana Grotte, Italy; ilenia.saponara@irccsdebellis.it (I.S.); miriam.cofano@irccsdebellis.it (M.C.); valentina.denunzio@irccsdebellis.it (V.D.N.); giuliano.pinto@irccsdebellis.it (G.P.); emanuela.caruso@irccsdebellis.it (E.A.C.); matteo.centonze@irccsdebellis.it (M.C.); 2 Animal Facility, National Institute of Gastroenterology IRCCS “Saverio de Bellis”, 70013 Castellana Grotte, Italy; giusy.bianco@irccsdebellis.it; 3 Histopathology Unit, National Institute of Gastroenterology IRCCS “Saverio de Bellis”, 70013 Castellana Grotte, Italy; raffaele.armentano@irccsdebellis.it; 4 Unit of Clinical Pathology, National Institute of Gastroenterology IRCCS “Saverio de Bellis”, 70013 Castellana Grotte, Italy

**Keywords:** liver fibrosis, ex vivo models, Hesperidin, fibrosis markers, liver inflammation, extracellular matrix proteins

## Abstract

Liver fibrosis is characterized by an excessive accumulation of extracellular matrix (ECM) proteins as a wound-healing response to chronic liver injury, leading to tissue scarring and organ dysfunction. Natural compounds, including phytonutrients and polyphenols, have been shown to exert protective effects by reducing profibrotic biomarkers in vitro and in vivo models. Here, we provide the first evidence that the polyphenol hesperidin (HE) can counteract the onset of fibrotic responses in an ex vivo mouse liver fibrosis model induced by Transforming Growth Factor-β1 (TGF-β1) (5 ng/mL). Notably, HE drives early ECM remodeling in the fibrotic mouse liver tissue. Fibrosis-related parameters were assessed at both the transcriptional and translational levels after treatment with HE at increasing concentrations of 50, 75, and 100 µg/mL. Interestingly, HE at 75 µg/mL exerted the strongest beneficial effect, significantly decreasing the gene expression of *α-SMA*, *SERPINH-1*, *FN-1*, *VIM* and *COL1A1* and counteracting the TGF-β1-induced upregulation of key fibrotic markers, including α-SMA, COL1A2, and VIM, reflecting its capacity to attenuate myofibroblast activation and ECM production and modulating membrane lipid peroxidation. Furthermore, HE inhibited SMAD2 phosphorylation, suggesting that its antifibrotic activity may involve the modulation of the TGF-β/SMAD signaling pathway. Moreover, it promoted an anti-inflammatory response, due to a decrease in IL-1β and IL-6 expression. Our study highlights the potential of the ex vivo model as a platform for evaluating the antifibrotic efficacy of natural molecules, and it suggests significant translational implications and new opportunities for developing innovative therapeutic strategies.

## 1. Introduction

Liver fibrosis represents the liver’s wound-healing response to chronic injury, and it is considered a common pathological feature of chronic viral hepatitis, non-alcoholic fatty liver disease, alcoholic liver disease, and cholestatic or autoimmune disorders [1]. It is driven by an imbalance between the production and degradation of ECM proteins. Progressive ECM accumulation gradually disrupts the normal liver architecture, and it leads to cirrhosis in advanced stages, increasing the risk of hepatocellular carcinoma (HCC) [2,3]. In experimental and clinical research, liver fibrosis is commonly assessed on histological sections using hematoxylin–eosin (H&E) or classical histochemical stains, such as Masson’s trichrome and Sirius Red, which reveal collagen accumulation and architectural distortion. However, conventional methods may not fully capture fibrillar extracellular matrix deposition, leading to an underestimation of fibrosis [4]. More specific techniques, including picrosirius red [5] and reticulin staining [6], are therefore frequently requested by hepatologists as a second-line approach to achieve a more accurate evaluation of collagen distribution and connective tissue organization in progressive liver diseases. In particular, reticulin-silver staining highlights the fine network of reticular fibers that maintain the hepatic architecture, offering critical insight into early structural alterations of ECM.

Hepatic stellate cells (HSCs), predominantly located in the space of Disse between hepatocytes and sinusoidal endothelium, are the main effectors of hepatic fibrogenesis [7]. In their quiescent state (qHSCs), they store vitamin A in lipid droplets. After fibrogenic stimulation, HSCs undergo transdifferentiation, assuming a myofibroblast-like phenotype, characterized by a marked upregulation of ECM proteins, such as type I collagen (COL1A) and Fibronectin-1 (FN-1), and an upregulation of α-smooth muscle actin (α-SMA) expression [8]. Activated HSCs secrete pro-inflammatory cytokines and chemokines (TNF-α, IL-1β, IL-6), facilitating the recruitment and activation of immune cells, including macrophages and T lymphocytes, thereby potentiating and sustaining the fibrogenic cascade [9].

At the molecular level, TGF-β1 is a key fibrogenic stimulus that, binding to its receptor, induces the phosphorylation of SMAD2 and SMAD3 (p-SMAD2/3), which then form complexes with SMAD4 and translocate into the nucleus to regulate the transcription of profibrogenic genes [10].

In recent years, precision-cut liver slices (PCLSs) have been increasingly recognized as reliable and physiologically relevant models for investigating liver fibrosis and preclinical assessment of antifibrotic therapies [11,12,13]. PCLS preserves the multicellular architecture of the liver and maintains both cell–cell and cell–matrix interactions [14,15]. This system captures the complex interplay between hepatocytes, endothelial cells, stellate cells, and resident immune populations in a physiologically relevant, fibrotic environment [14,16]. PCLS allows for the study of immune-mediated amplification of fibrosis through pro-inflammatory cytokines and chemokines, which drive stellate cell activation and myofibroblast differentiation [17]. Compared to traditional in vitro models, PCLSs more closely resemble the in vivo conditions and provide a valuable platform for investigating fibrogenic mechanisms and evaluating potential antifibrotic therapies.

HE is a natural flavanone glycoside present in high concentrations in citrus fruits and has been extensively studied for its hepatoprotective properties [18]. These beneficial effects are attributed to its antioxidant [19], anti-cytotoxic [20], anti-inflammatory [18], antifibrotic [21], and antitumor activities [20,22], making HE a strong candidate for the prevention and treatment of chronic liver diseases. At the molecular level, the antifibrotic activity of HE is closely associated with the modulation of the TGF-β1/Smad signaling pathway, a key regulator of HSC activation and ECM deposition. In parallel, HE exhibits pronounced anti-inflammatory activity, inhibiting nuclear factor kappa B (NF-κB) activation and suppressing the production of pro-inflammatory cytokines, including IL-1β, IL-6, and interferon-gamma (IFN-γ), while simultaneously enhancing the expression of anti-inflammatory mediators such as interleukin-10 (IL-10).

In particular, polyphenols are particularly known for their antioxidant capacity, which includes the ability to neutralize various reactive oxygen species (ROS), reduce the propagation of oxidative reactions, and preserve the structural integrity of cell membranes by limiting membrane lipid peroxidation [23]. Lipid peroxidation, which is induced by oxidative stress, activates inflammatory pathways, as the pro-inflammatory cytokine production, which contribute to the overregulation of ROS [24]. These processes can be investigated by studying membrane phospholipids with lipidomic analysis.

In our previous in vitro experimental settings involving hepatic stellate cells (LX-2) and hepatocytes (Hepa-RG), HE treatment downregulated the expression of critical fibrogenic markers, including *α-SMA* and *COL1A1*. This inhibitory effect reduces ECM deposition and effectively prevents fibrosis progression [25,26].

In light of our previous findings, in the current study we aimed to analyze the beneficial effects of HE in counteracting liver fibrosis in healthy thin transversal hepatic slices treated with TGF-β1. Specifically, we evaluated the ability of this polyphenol to modulate the expression of profibrotic markers during the early stages of liver fibrosis using a relevant ex vivo model.

## 2. Results

### 2.1. Viability of Hepatic Slices

**Figure 1:**
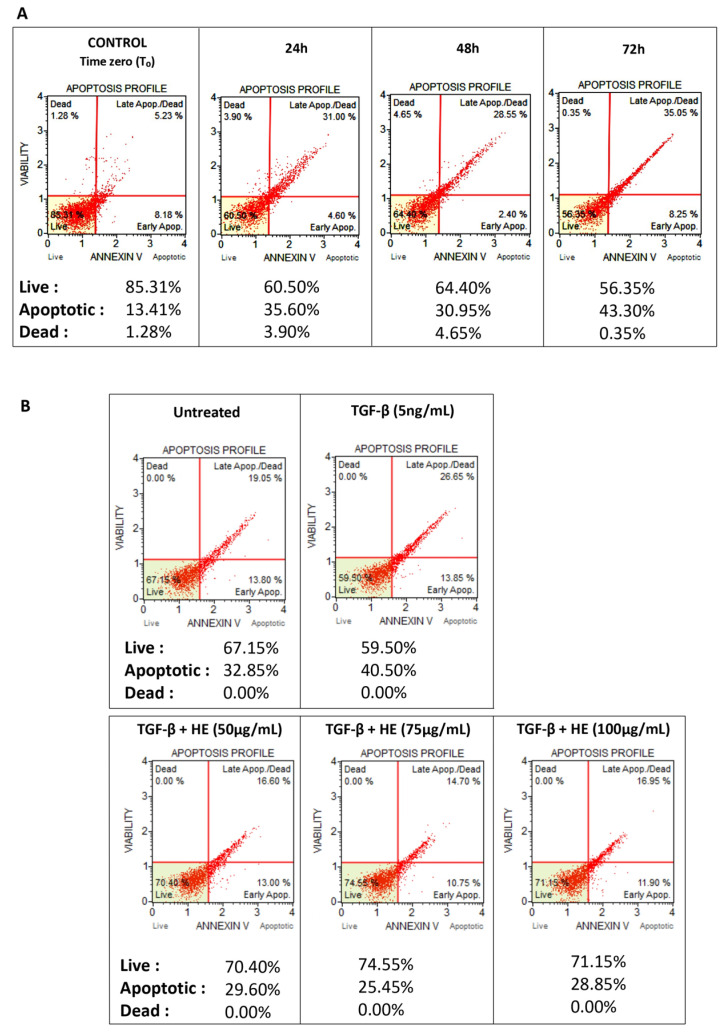
Hepatic slices viability assessed directly at T_0_ and after 24, 48, and 72 h (**A**) and under experimental treatment conditions (**B**) of culture using the Muse Annexin V & Dead Cell Kit. The representative dot plots of flow cytometry are presented as CONTROL (TIME ZERO-T_0_), 24 h, 48 h, and 72 h. Co-detection of Annexin V (Pto-L-ser) and 7-Amino-Actinomycin D (7-AAD) indicate apoptosis or necrosis, respectively. The red dots indicate cell populations within a sample. Cells in the live quadrant (bottom left) are viable, non-apoptotic and negative for Annexin V and 7-AAD. Cells in the early apoptotic quadrant (bottom right) are positive Annexin V only, while the late apoptotic/dead quadrant (top right) represents cells that are positive for annexin V and 7-AAD. The dead quadrant (top left) represents mostly nuclear debris and are cells positive for 7-AAD only.

To assess the viability of hepatic slices, Annexin V/dead cell assays were performed directly on fresh healthy slices (T_0_) and after 24, 48, and 72 h of culture (Figure 1A). No significant differences in slice viability were observed at 24 h (LIVE: 60.50%) and 48 h (LIVE: 64.40%), when normalized to T_0_ (85.31%). However, a slight initial decrease of ~20% in the proportion of live (LIVE) cells was detected at 24 h, which remained relatively stable up to 48 h. At 72 h, the percentage of live cells decreased by ~30%. Based on these results, subsequent experiments were limited to 48 h of culture. Furthermore, assessment of hepatic slices viability under the experimental conditions revealed no cytotoxic effects following HE treatment at any of the concentrations tested. Viability remained stable, displaying a slight HE-induced increase relative to only TGF-β-treated slices (Figure 1B). Viability analyses using Crenigagestat (5 µM) as a positive control are provided in the Supplementary Materials (Figure S1).

### 2.2. Effect of HE on TGF-β1-Induced Expression of Fibrosis and Inflammation-Related Genes

**Figure 2:**
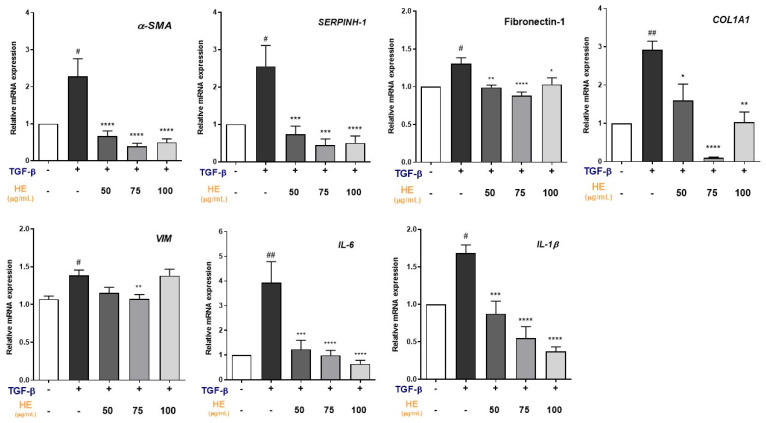
The effect of increasing doses of HE (50, 75, and 100 µg/mL) on gene expression of fibrosis markers (*α-SMA*, *COL1A1*, *VIM, SERPIN-1*, *Fibronectin-1*) and inflammatory cytokines (*IL-6* and *IL-1β*) in PCLS treated for 48 h with TGF-β1 (5 ng/mL). All data reported in each panel are expressed as the mean ± SEM from three independent experiments (*n* = 3 for each condition). Statistical analyses: one-way ANOVA, Dunnet’s post hoc analysis: TGF-β co-treated HE compared to TGF-β treated slices, (* *p* < 0.05, ** *p* < 0.01, *** *p* < 0.001, **** *p* < 0.0001) and #: Untreated compared to TGF-β treated slices, (# *p* < 0.05, ## *p* < 0.01).

To investigate whether HE could modulate the pro-fibrotic and pro-inflammatory responses in TGF-β1 (5 ng/mL)-treated hepatic slices, the expression of specific related markers was analyzed (Figure 2).

RT-qPCR analysis revealed that TGF-β1 significantly upregulated the expression of the myofibroblast differentiation markers *α-SMA* (*p* < 0.05) and *SERPINH-1* (*p* < 0.05) relative to the untreated controls. Moreover, the mRNA levels of ECM components, including *FN-1* (*p* < 0.05) and type I collagen *COL1A1* (*p* < 0.01), were markedly elevated following TGF-β1 stimulation compared to those in the control group. Given that the fibrotic phenotype was maintained in ex vivo TGF-β1 (5 ng/mL) treated hepatic slices, we analyzed the possible modulatory effects of HE. Notably, the expression of α*-SMA*, *SERPINH-1*, *FN-1*, *VIM* and *COL1A1* was significantly inhibited after 48 h of HE treatment at all tested concentrations, with the strongest inhibitory effect observed at 75 µg/mL for each marker. In addition, TGF-β1 exposure induced a pro-inflammatory response, as indicated by the strong upregulation of *IL-6* and *IL-1β* mRNA expression, which was significantly attenuated by HE in a dose-dependent manner compared to hepatic slices treated with TGF-β1 alone.

### 2.3. Effect of HE on Protein Expression of Key Mediators Involved in Fibrogenic Processes

**Figure 3:**
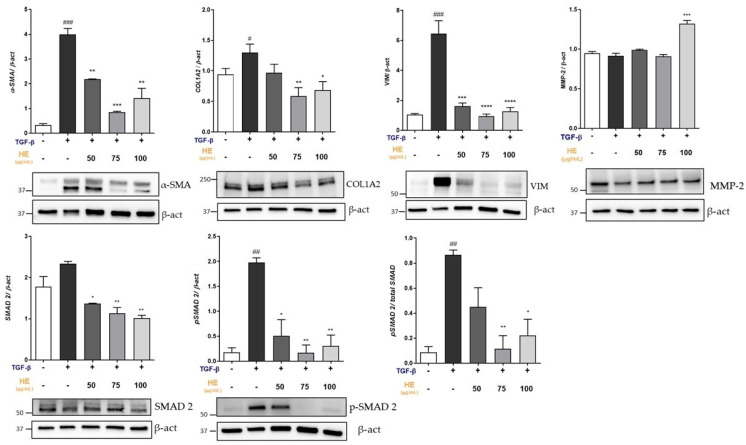
Effect of HE on α-SMA, COL1A2, MMP-2 and VIM protein expressions and the p-SMAD2/SMAD2 ratio in TGF-β-treated PCLS was measured by Western blot, and β-ACTIN was used as a loading control. Representative Western blot bands are shown. All data reported in each panel are expressed as the mean ± SEM from three independent experiments (*n* = 3 for each condition). Statistical analyses: one-way ANOVA Dunnet’s post hoc analysis: TGF-β co-treated HE compared to TGF-β treated slices, (* *p* < 0.05, ** *p* < 0.01, *** *p* < 0.001, **** *p* < 0.0001) and #: Untreated compared to TGF-β treated slices, (# *p* < 0.05, ## *p* < 0.01, ### *p* < 0.001).

At the translational level, TGF-β1 (5 ng/mL) induced a fibrotic response, as evidenced by a significant increase in α-SMA, COL1A2, and VIM protein levels compared to the control group (Figure 3). Interestingly, 48h of HE treatment counteracted these effects, significantly reducing COL1A2, α-SMA, MMP-2 and VIM protein expression, with detectable effects at 50 µg/mL, and more pronounced inhibition observed at 75 µg/mL.

To further elucidate the molecular mechanisms underlying the antifibrotic action of HE, we investigated the involvement of the TGF-β/SMAD signaling pathway. Analysis of SMAD2 protein expression revealed a marked increase in phosphorylation following TGF-β1 treatment. Notably, HE at 75 µg/mL significantly attenuated TGF-β1-induced SMAD2 phosphorylation, suggesting that HE may exert its antifibrotic effects, through modulation of the canonical TGF-β/SMAD pathway. TGF-β-induced fibrotic hepatic slices were treated with Crenigagestat (5 µM, CRE) as a positive control, and multiple protein markers were subsequently analyzed (Figure S2).

### 2.4. HE Modulates Inflammatory and Profibrotic Cytokine Release in TGF-β-Treated Hepatic Slices

**Figure 4:**
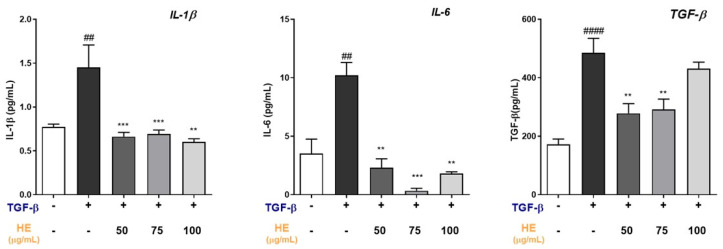
Quantification of IL-1β, IL-6, and TGF-β levels in culture supernatants of hepatic slices untreated or treated with TGF-β (5 ng/mL) for 48 h, in the absence or presence of HE (50, 75, and 100 µg/mL). Cytokine concentrations were measured by ELISA. All data reported in each panel are expressed as the mean ± SEM from three independent experiments (*n* = 3 hepatic slices for each condition). Statistical analyses: one-way ANOVA Dunnet’s post hoc analysis: TGF-β co-treated HE compared to TGF-β treated slices (** *p* < 0.01, *** *p* < 0.001) and #: Untreated compared to TGF-β treated slices (## *p* < 0.01, #### *p* < 0.0001).

Exposure of hepatic slices to TGF-β (5 ng/mL) for 48 h resulted in a significant increase in the release of the pro-inflammatory cytokines IL-1β and IL-6, as well as the profibrotic cytokine TGF-β, compared with untreated controls (Figure 4). Treatment with HE markedly attenuated TGF-β-induced cytokine secretion in a concentration-dependent manner. IL-1β levels were significantly reduced at all tested HE concentrations (50, 75, and 100 µg/mL), reaching values comparable to or lower than those observed in untreated slices. Similarly, HE treatment significantly suppressed IL-6 release, with the strongest inhibitory effect observed at 75 µg/mL. In contrast, while TGF-β stimulation robustly increased endogenous TGF-β release, HE treatment partially but significantly reduced TGF-β levels at 50 and 75 µg/mL. The highest HE concentration (100 µg/mL) showed a weaker inhibitory effect, with TGF-β levels remaining elevated compared with lower HE doses.

### 2.5. Protective Role of HE on TGF-β-Induced Liver Injury Assessed by H&E and Silver-Reticulin Impregnation Staining

**Figure 5:**
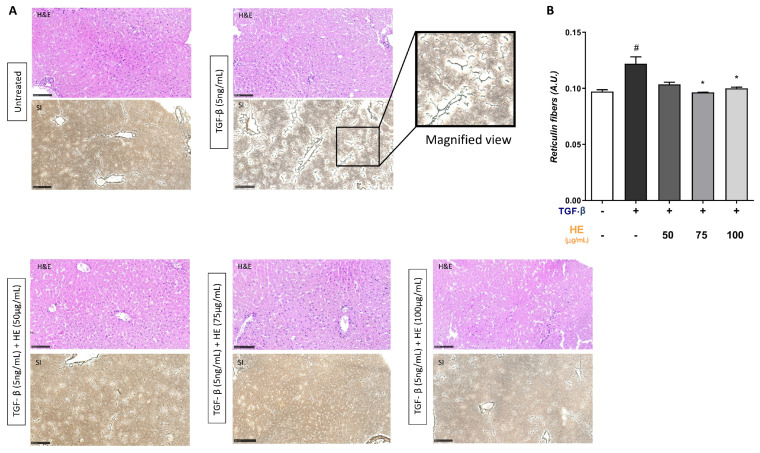
H&E staining and reticulin-silver impregnation (SI) of hepatic sections after 48 h treatment with TGF-β1 (5 ng/mL) and co-treatment with HE at 50, 75, and 100 μg/mL In the panel, TGF-β1-treated hepatic slices showed dark-staining fiber bundles markedly infiltrating the hepatic parenchyma, clearly visible at higher magnification. In the co-treatment conditions with HE (50, 75, and 100 μg/mL), a reduction in reticulin structure and the presence of smaller reticular fiber fragments can be observed (**A**). Reticulin fibers were quantified by ImageJ software (version 1.54g), normalized to image area (**B**). Scale bars, 100 μm. Each experiment was performed at least three times. Statistical analyses: one-way ANOVA Dunnet’s post hoc analysis: TGF-β co-treated HE compared to TGF-β treated slices (* *p* < 0.05) and #: Untreated compared to TGF-β treated slices (# *p* < 0.05).

To evaluate the preservation and structural integrity of hepatic slices, H&E staining was performed. As shown in Figure 5, hepatic slices maintained well-preserved lobular architecture under all experimental conditions after 48 h of culture, with only minor structural alterations. In parallel, silver staining for reticular fibers (composed mainly of collagen types III and IV) was used to investigate the pattern of reticulin fiber remodeling in the process of liver fibrosis. Following TGF-β1 treatment, a marked accumulation and disorganization of reticular fibers were observed, characterized by aberrant branching and infiltration into the lobular parenchyma extending from the portal spaces, as shown in the magnified view. Remarkably, co-treatment with HE, in particular at 75 µg/mL, substantially restored the regular reticulin framework, significantly reducing excessive fiber density and abnormal branching. Images of reticulin impregnation and H&E staining of the positive control (CRE) are provided in the Supplementary Materials (Figure S3).

### 2.6. Effect of HE on the Lipidomic Profile in Hepatic Slices

Table 1 shows the percentage value of fatty acids in hepatic slices treated with TGF-β1 (5 ng/mL) alone and with HE at different concentration after 48 h using a gas chromatography method. As can be observed, TGF-β1 induces an increase in the peroxidation index (PI) which is reversed by cotreatment with HE for 48 h. In particular, with HE 50 μg/mL the PI is reduced to lower values than those of the untreated slices.

This reduction was due to a significant increase in monounsaturated fatty acids (MUFAs), particularly oleic acid, especially at 50 μg/mL. At the same concentration, a significant reduction in stearic acid, dihomo γ-linoleic acid (DGLA, C20:3n6), arachidonic acid (AA, C204:n6) eicosapentaenoic acid (EPA, C20:5n3) and docosahexaenoic acid (DHA, C22:6n3) was also observed. While comparing HE 75 μg/mL with PCLS treated with TGF-β1 (5 ng/mL), a decrease in DHA and stearoyl-CoA Desaturase-1 (SCD1) was detected.

## 3. Discussion

In this study, we induced fibrosis by stimulating healthy mouse liver slices with 5 ng/mL of TGF-β for 48 h, and for the first time, we demonstrated that HE can counteract the onset of fibrotic responses in liver tissue, using an ex vivo model.

Recently, PCLS models have emerged as a relevant ex vivo approach for investigating liver fibrosis and evaluating the efficacy of experimental compounds in a pathophysiological environment [12,27,28,29]. Compared with other in vitro models, such as three-dimensional co-culture platforms and more complex systems like liver organoids, these ex vivo models retain the native tissue architecture and preserve the tissue’s biomechanical properties, sustaining the cell-extracellular matrix interactions that are critical for regulating fibrogenic pathways [14]. In vivo models allow for the study of complex systemic responses, including immune cell recruitment and activation, vascularization, systemic metabolism, and the temporal dynamics of tissue remodeling, and they are therefore regarded as the gold standard for the study of liver fibrosis.

Conversely, ex vivo models offer several key advantages, such as a substantial reduction in animal use to enable rapid testing of multiple compounds, and improve reproducibility. PCLSs also allow simultaneous evaluation of efficacy, toxicity, and metabolism in native liver tissue, overall enhancing the predictive value in preclinical studies.

In the experimental setting, to study the molecular mechanisms in advanced clinical stages of liver fibrosis, PCLSs obtained from liver biopsies of patients affected by liver disease fibrosis or from animal models subjected to in vivo induction of fibrosis or cirrhosis are used [30].

To investigate the early molecular mechanisms involved in liver fibrosis, it is advantageous to use healthy hepatic slices, in which a fibrotic phenotype is experimentally induced through short-term (24–48 h) exposure to fibrogenic agents such as ethanol [31], bile acids [32], carbon tetrachloride (CCl_4_) [33], or thioacetamide [34]. In this scenario, Sadasivan et al. [35] developed a fibrosis model using PCLSs treated with a fibrogenic cocktail composed of TGF-β, platelet-derived growth factor, lysophosphatidic acid, sphingosine-1-phosphate, lipopolysaccharide, and palmitate. More importantly, Westra et al. [11] and Huang et al. [29] explored the antifibrotic activity of various compounds on fibrotic PCLSs derived from rats subjected to bile duct ligation (BDL). Hence, several studies have provided a valuable overview of the genes and gene products (α-SMA, HSP47, COL1A1, TIMP-1, MMP-7, and MMP-1) that are dysregulated in preclinical models of fibrotic liver diseases and/or in fibrotic human liver tissues, thereby offering relevant efficacy endpoints to assess the antifibrotic potential of experimental compounds [17].

TGF-β is a key cytokine driving the pathogenesis of liver fibrosis by inducing the activation of HSCs and promoting the deposition of ECM components. The profibrotic effect of TGF-β has been demonstrated both in vivo, through enhanced collagen deposition in mouse models [36,37,38], and in vitro, significantly upregulating the expression of several pro-fibrogenic markers in LX-2 cells, including α*-SMA*, *FN-1*, *MMP-2*, *MMP-9*, *TIMP-1*, and *TIMP-2* [39]. To date, the hepatoprotective role of natural compounds, such as polyphenols, is increasingly recognized for their potential to improve liver function and mitigate pathological processes associated with hepatic dysfunction.

In our previous studies, we demonstrated the antifibrotic activity of HE in hepatic stellate cells (LX-2) activated by TGF-β or activated with a mixture of NAFLD-promoting agents (NPAs) to mimic the pathophysiological environment observed in NAFLD/NASH patients. These studies have demonstrated that HE interferes with key signaling pathways mediating fibrotic activation in hepatic stellate cells [25,26].

Based on these results, we investigated the effects of HE in an ex vivo model, given that, to our knowledge, a comprehensive evaluation of this natural compound has not yet been conducted using an ex vivo model. The TGF-β-induced fibrotic model in PCLSs does not fully reproduce the multifactorial nature of chronic liver fibrosis in vivo, as it lacks systemic immune interactions, metabolic alterations, and pharmacokinetic considerations, and it is limited by a short culture timeframe. Nevertheless, it provides a complementary platform bridging in vitro assays and in vivo studies. Therefore, the results of this study should be interpreted as preliminary preclinical evidence, useful for the identification and selection of antifibrotic candidates, such as the promising natural compounds including the HE evaluated in this work.

In this study, we demonstrated that treatment of PCLSs with TGF-β1 led to upregulation of *COL1A1*, *α-SMA*, *SERPINH-1*, *VIM* and *FN-1* gene expression, resulting from the activation of HSCs, not ruling out, in our ex vivo model, the contribution of fibroblast-like cells (i.e., portal fibroblasts), although HSCs remain the principal ECM-producing cells [40]. Moreover, the increased phosphorylation of the downstream signaling protein SMAD2 confirmed that TGF-β1 stimulation triggered a fibrotic response, contributing to the initiation of early-stage fibrosis.

Interestingly, co-treatment with HE effectively reversed the increase in these markers at all tested concentrations. In this study, we demonstrated that HE significantly downregulated the gene expression of interleukins *IL-1β* and *IL-6* at all tested concentrations in a dose-dependent manner. Also, HE effectively attenuated the protein expression of α-SMA, MMP-2, COL1A2 and VIM, markers involved in ECM remodeling, as well as downregulated p-SMAD2. It should be noted that COL1A1 was measured by qPCR to assess early transcriptional changes, whereas COL1A2 protein levels reflect collagen fibril deposition in TGF-β-treated hepatic. Interestingly, we found that co-treatment with HE led to a marked reduction in the endogenous release of the pro-fibrotic cytokine TGF-β, a key reporter of treatment efficacy, quantified by ELISA. Our results show that HE markedly suppressed the gene expression of the pro-inflammatory interleukins IL-1β and IL-6 in a clear dose-dependent manner. Importantly, in the absence of cytotoxicity, the apparent mismatch between the mRNA levels of IL-6 and IL-1β and their actual extracellular secretion, along with the rebound observed at the highest concentration (100 μg/mL), likely reflects a combination of factors. These may include a saturation of HE’s modulatory capacity, compensatory cellular mechanisms aimed at restoring homeostasis, or delayed feedback loops that temporarily counteract the initial suppression.

Downregulation of pro-inflammatory cytokines by polyphenols reduces oxidative stress [23], creating a microenvironment less permissive to collagen synthesis and maturation. These synergistic mechanisms likely account for the effective suppression of COL1A1 expression observed in TGF-β1-treated PCLSs following HE treatment. In addition, HE can also modulate the tissue lipidomic profile. Lipid peroxidation, which is triggered by oxidative stress, activates inflammatory pathways that increase the production of ROS. The literature indicates that, in pathological conditions, lipid peroxidation is a cellular response mechanism to oxidative stress. The fatty acids most susceptible to peroxidation are those with a high number of double bonds, such as PUFAs present in cell membranes [24]. This study demonstrates that HE exerts their antioxidant effects by modulating the tissue fatty acids profile, reducing the levels of membrane peroxidation.

In ECM composition, reticular fibers represent a key structural component, they are composed primarily of type III collagen, in association with other collagen types, such as type V collagen, as well as glycoproteins and proteoglycans/glycosaminoglycans. Silver staining was employed to closely examine alterations in the reticular fiber network in the context of experimentally induced liver fibrosis by TGF-β1 and to assess the modulatory effects of HE treatment. The results demonstrated that HE is capable of modulating the structure of reticular fibers, markedly reducing both branching complexity and fiber density, and promoting the presence of thinner, smaller fiber fragments, suggesting a protective effect of HE against TGF-β1-induced fibrotic ECM remodeling. HE emerges as a promising nutraceutical and pharmacological agent for liver protection, owing to its pleiotropic profile that combines antioxidant, anti-inflammatory, lipid-regulating, and hepatoprotective effects.

However, a limitation of this model is the relatively short culture period (48–72 h), which may not be sufficient to detect post-translational events or long-term fibrogenic processes. As in other ex vivo models, circulating immune cells that actively contribute to hepatic fibrogenesis are absent due to the lack of blood flow, although PCLSs preserve the native hepatic vascular architecture. At the translational level, the effective concentrations of HE have been tested in an ex vivo model, which lacks systemic metabolism, absorption, and clearance. As a result, the observed effective doses may not be directly translatable to clinical settings.

Taken together these data suggest that HE was able to counteract the onset of fibrotic responses, in this ex vivo model, as well as to negatively modulate the inflammatory response. Moreover, our study reveals promising results, highlighting the potential of the ex vivo model as a platform for evaluating the antifibrotic efficacy of natural molecules, with significant translational future implications and new opportunities for the development of innovative therapeutic strategies.

## 4. Materials and Methods

### 4.1. Hepatic Slices Preparation and Experimental Treatment

Liver samples were obtained from mice used in another experimental protocol approved by the Italian Ministry of Health, in accordance with national and international animal welfare regulations (Italian Legislative Decree No. 26/2014). Therefore, no animals were sacrificed specifically for this study. All liver samples were collected from C57BL/6J mice immediately after anesthesia with 2% isoflurane (*v*/*v*), followed by cervical dislocation. Liver tissues were obtained at the end of the previous procedure and subsequently used to prepare thin transverse hepatic slices (250–300 μm) [41,42].

The hepatic slices were incubated individually in 4 mL of cold Dulbecco’s Modified Eagle’s Medium (DMEM; Thermo Fisher Scientific, MI, Italy) previously supplemented with 10% heat-inactivated Fetal Bovine Serum (FBS) and 1% antibiotic-antimycotic solution (10,000 U/mL penicillin, 10,000 μg/mL streptomycin, and 25 μg/mL Gibco Amphotericin B).

Subsequently, the slices were placed on an 8 µm-pore Transwell insert (Corning, Glendale, AZ, USA) in a 6 well plate. All slices were cultured in 2 mL of fresh growth medium in the absence of FBS at 37 °C in a humidified incubator with 5% CO_2_, shaking at 90 rpm. The medium was refreshed every 24 h. For the fibrotic-inducing experiments the slices were incubated with the pro-fibrotic compound TGF-β (cod. HY-P70543 MedChemExpress, Monmouth Junction, NJ, USA) for 48 h. To establish the effect of HE, the PCLSs were treated with three different concentrations (50, 75, and 100 µg/mL) of HE (cod. H5254 Sigma-Aldrich, St. Louis, MO, USA). Briefly, the culture medium was removed and replaced with fresh medium containing HE for a 2 h pre-treatment. Subsequently, 5 ng/mL of TGF-β was added in the presence of HE treatment (50, 75, and 100 µg/mL) for 48 h. Control slides were treated with an equivalent volume/volume (*v*/*v*) concentration of the vehicle present at the highest treatment concentration of HE (100 µg/mL) Dimethyl Sulfoxide (DMSO; cod. D2438, Sigma Chemical, St. Louis, MO, USA). HE treatment concentrations (50, 75, and 100 µg/mL) were prepared from the 10,000 µg/mL concentration by diluting with culture medium. All experiments were performed in triplicate (technical replicates) using liver tissue from 3 to 6 mice.

### 4.2. Viability Assay

Preliminary, tissue specimens were subjected to enzymatic and mechanical digestion (Sigma-Aldrich, St. Louis, MO, USA) solution with 50–200 U/mL collagenase Type IV (ThermoFisher Scientific, Waltham, MA, USA), 3 mM CaCl_2_ and 0.5 mM MgCl_2_, and Antibiotic–Antimycotic (ThermoFisher Scientific, Milan, Italy) at 37 °C under gentle rotation for 2 h or more. At the end of this step, homogenized tissue specimens were centrifuged at 500× *g* for 10 min and resuspended pellet in 1 mL of DMEM for the following cell viability assessment. For quantitative analysis of live, early/late apoptotic, and dead cells we used the Muse Annexin V/Dead Cell Assay Kit (Luminex Corporation, Austin, TX, USA) on a Muse Cell Analyzer. Briefly, the assay utilized Annexin V to detect phosphatidylserine on the external membrane of apoptotic cells. The fluorescent signal emitted by dye-conjugated antibodies was detected by flow cytometry technology (Muse Cell Analyzer, Luminex Corporation, Austin, TX, USA). 7-AAD cell dead marker was also used. Cells were analyzed according to the manufacturer’s instructions.

### 4.3. Histological Staining

Liver slices were fixed overnight in 4% paraformaldehyde at 4 °C, embedded in paraffin, and sectioned at 4 μm thickness. Hematoxylin and eosin (H&E) staining (Bio-Optica, Milan, Italy, catalog number 05-06002E) and a silver impregnation-based kit (Bio-Optica, Milan, Italy, catalog number 04-040801) for reticulin staining were performed separately to evaluate tissue architecture and to identify reticular fibers. All histological slides were digitally scanned using a NanoZoomer^®^ S20 digital slide scanner (Hamamatsu, Herrsching am Ammersee, Germany), and five images were randomly acquired per section (at ×200 magnifications with scale bar 100 µm). Each experiment was performed at least three times.

### 4.4. Gene Expression Analysis by Real-Time Quantitative Reverse Transcription PCR

Hepatic slices were homogenized in Trizol reagent (Thermo Fisher Scientific, Waltham, MA, USA) using a Tissue Lyser II homogenizer (QIAGEN, Hilden, Germany; Cat. No. 85300), and total RNA was extracted according to the manufacturer’s instructions. RNA was precipitated with ethanol 75%, and resuspended in nuclease-free water. cDNA was obtained by RNA reverse transcription from 1 µg of RNA using iScript Adv cDNA kit for RT-qPCR (Bio-Rad Laboratories, San Francisco, CA, USA). cDNA was then analyzed by qPCR with the SYBRGreenER™ qPCRSuperMix Universal (Thermo Fisher Scientific, Milan, Italy). RNA expression level was determined using the comparative cycle threshold (Ct) method, where the amount of target cDNA was normalized to housekeeping genes β-ACTIN cDNA. When hepatic slices were treated with HE, the amount of target cDNA was normalized to housekeeping genes and to the TGF-β (5 ng/mL) treated hepatic slices cDNA (2^−ΔΔCt^). Primers used for the qPCR step are presented in Table 2.

### 4.5. Western Blot Analysis

Hepatic slices were lysed using a tissue homogenizer (TissueLyser II, QIAGEN, Hilden, Germany; Cat. No. 85300) in RIPA buffer (Sigma-Aldrich, St. Louis, MO, USA) supplemented with the Halt Protease and Phosphatase Inhibitor (ThermoFisher Scientific, Waltham, MA, USA).

The lysate was recovered and centrifuged at 13,000 rpm for 30 min at 4 °C, and the supernatant was collected and used for total protein quantification by a standard Bradford assay (Bio-Rad Laboratories, San Francisco, CA, USA). An equal amount of protein (30 μg) was separated in 4–15% Tris-glycine sodium dodecyl sulfate-polyacrylamide gel (Bio-Rad Laboratories, San Francisco, CA, USA). Membranes were incubated with gentle shaking, with the following primary antibodies: *COL1A2* (Rabbit, pAb #96723, 1:500, Abcam, Cambridge Biomedical Campus, Cambridge, UK), *SMAD2* (Rabbit, mAb #5339, 1:1000, Cell Signaling Technology, Danvers, MA, USA), *pSMAD2* (Rabbit, mAb #280888, 1:1000, Abcam, Cambridge Biomedical Campus, Cambridge, UK), *VIMENTIN* (Rabbit mAb #5741, 1:1000, Cell Signaling Technology, Danvers, MA, USA), α*-SMA* (Rabbit mAb #19245, 1:500, Cell Signaling Technology, Danvers, MA, USA) and MMP-2 (Rabbit AB86607, Abcam Cambridge Biomedical Campus, Cambridge, UK). The *COL1A2*, *pSMAD2* and α-*SMA* antibodies were diluted in 5% *w/v* no fat dry milk, 1× Tris-Buffered Saline (TBS), and 0.1% Tween^®^ 20. *SMAD2* and *VIMENTIN* antibodies were diluted in 5% *w*/*v* BSA, 1× TBS, and 0.1% Tween^®^ 20. Experiments were carried out in triplicate. After overnight incubation of primary antibodies, anti-rabbit or anti-mouse secondary antibody (1:5000 Bio-Rad Laboratories, San Francisco, CA, USA), diluted in 5% *w*/*v* no fat dry milk, 1× TBS, and 0.1% Tween^®^ 20, was incubated for one hour with gentle shaking. The chemiluminescence signal from proteins was revealed using Clarity Western ECL Substrate or Clarity Max Western Enhanced Chemiluminescence (ECL) Substrate (Bio-Rad Laboratories, San Francisco, CA, USA) and analyzed using the chemiluminescence detection system ChemiDoc XRS (Bio-Rad Laboratories, San Francisco, CA, USA). The relative density of the bands was calculated using the ImageLab software 5.2.1 and the proteins detected were normalized against the expression of the housekeeping gene β-actin (Mouse, mAb #3700, 1:1000, Cell Signaling Technology, Danvers, MA, USA).

### 4.6. Fatty Acid Extraction, Preparation of Fatty Acid Methyl Esters, and Fatty Acid Quantification from Tissue Samples

Fatty acid extraction from tissues was performed using the Fatty Acid Extraction Kit, Low Standard (Sigma-Aldrich, St. Louis, MO, USA) according to the manufacturer’s instructions. Briefly, 20 mg of treated tissues were homogenized with 3 mL of extraction solvent, then 500 μL of aqueous buffer was added, vortexed, and placed in a syringe to elute the lipids. The eluted lipids were dried and esterified with a methanolic potassium hydroxide solution. After sonication, 1 mL of hexane and 1 mL of distilled water were added to the extracted mixture. The upper portion containing the lipids was transferred to a new tube and dried. The esterified lipids were reconstituted with 500 μL of hexane and analyzed by gas chromatography using a gas chromatography apparatus with an autosampler, split/splitless injector, FID detector, and hydrogen gas generator (Thermo Fisher Scientific, Milan, Italy). A BPX 70 SGE Analytical Science capillary column, P/N SGE054623, 60 m × 0.25 mm ID, BPX70 0.25 μm (SGE Europe Ltd., Milton Keynes, UK) was used, and the injected amount was 2 μL in splitless mode (split flow rate 50 mL/min, splitless time 1 min). Quantification of fatty acid methyl esters was performed using a standard mix (Supelco 37-Component FAME Mix, Sigma-Aldrich, Milan, Italy). Of the 37 fatty acids detected, only a few representatives of the three families SFAs, MUFAs and PUFAs were chosen. In particular, for SFAs, palmitic acid (C16:0) and stearic acid (C18:0); for MUFAs, palmitoleic acid (C16:1n7) and oleic acid (C18:1n9), vaccenic acid (C18:1n7); for PUFAs, linoleic acid (C18:2n6), GLA (C18:3n6), DGLA (C20:3n6), AA (C20:4n6), EPA (C20:5n3) and DHA (C22:6n3). Considering these fatty acids, two indices were calculated: the PI (%MUFAs/0.025) + (%LA) + (%DGLA/2) + (%AA/4) + (%EPA/6) + (%DHA/8) and the SCD1 (oleic acid/stearic acid) desaturase enzyme index.

### 4.7. Enzyme-Linked Immunosorbent Assay (ELISA)

The concentrations of IL-1β, IL-6, and TGF-β released into the culture medium were quantified in untreated hepatic slices and in hepatic slices treated with HE (50, 75, and 100 µg/mL) and exposed to recombinant TGF-β (5 ng/mL) for 48 h. Cytokine levels were measured using enzyme-linked immunosorbent assays (ELISA) according to the manufacturers’ instructions. IL-1β was quantified using the Mouse IL-1β ELISA Kit (Ab197742, Abcam, Cambridge Biomedical Campus, Cambridge, UK), IL-6 using the DuoSet ELISA kit (DY206, R&D Systems, Minneapolis, MN, USA) and TGF-β using a high-sensitivity ELISA kit (DB100C, R&D Systems, Minneapolis, MN, USA). Absorbance was measured with a microplate reader, and cytokine concentrations were calculated from standard curves.

### 4.8. Statistical Analysis

Data were expressed as mean ± SEM, and all experiments were performed independently three times (n = 3). Statistical analyses were conducted using GraphPad Prism 8.0.1. One-way ANOVA with Dunnett’s post hoc correction for multiple comparisons was performed to assess differences in lipidomic profiles and to evaluate protein and gene expression levels between CTR and TGF-β-treated slices, as well as between TGF-β-treated slices and increasing concentrations (50, 75, and 100 µg/mL) of HE. Statistical significance was set at # *p* < 0.05, ## *p* < 0.01, ### *p* < 0.001, and #### *p* < 0.0001 for comparisons with CTR, and at * *p* < 0.05, ** *p* < 0.01, *** *p* < 0.001, and **** *p* < 0.0001 for comparisons among TGF-β-treated slices and HE treatments.

## 5. Conclusions

In conclusion, the ex vivo model preserves the native tissue architecture and cellular interactions, proved to be an effective experimental platform for investigating the effects of bioactive compounds. In our ex vivo mouse liver fibrosis model induced by TGF-β1 (5 ng/mL), HE exhibited antifibrotic effects by reducing the expression of key fibrogenic genes (*α-SMA*, *COL1A1*, *SERPINH-1*, *VIM* and *FN-1*) and by inhibiting the TGF-β/SMAD2 signaling pathway, indicating a negative modulation of the central molecular axis driving early-stage hepatic fibrosis progression. In parallel, HE exerted a pronounced anti-inflammatory effect by suppressing both the gene expression and extracellular levels of the pro-inflammatory cytokines IL-1β and IL-6 while also reducing endogenous TGF-β release. Together, these effects, combined with HE’s ability to modulate the tissue lipid profile, contribute to the establishment of a microenvironment that is less permissive to extracellular matrix deposition and remodeling.

## Figures and Tables

**Table 1:** Relative percentage of membrane fatty acid profile in hepatic slices.

**Treatment**					
TGF-β (5 ng/mL)	-	+	+	+	+
HE (μg/mL)	-	-	50	75	100
**Fatty Acid (%)**					
Palmitic acid (C16:0)	24.22 ± 1.51	23.32 ± 1.61	24.48 ± 1.33	22.34 ± 0.59	23.30 ± 0.20
Stearic acid (C18:0)	15.49 ± 1.24	15.46 ± 0.32	13.38 ± 0.46 *	17.07 ± 0.44	14.70 ± 0.17
Total SFAs	42.09 ± 3.19	40.93 ± 1.46	40.15 ± 1.29	41.53 ± 0.47	39.88 ± 0.50
Palmitoleic acid (C16:1n7)	0.37 ± 0.04	0.37 ± 0.03	0.35 ± 0.015	0.34 ± 0.03	0.36 ± 0.03
Oleic acid (C18:1n9)	12.99 ± 0.25	14.09 ± 0.27	15.56 ± 0.30 **	13.29 ± 0.07	14.14 ± 0.94
Vaccenic acid (C18:1n7)	2.585 ± 0.39	2.95 ± 0.15	2.54 ± 0.01	2.90 ± 0.01	3.08 ± 0.02
Total MUFAs	19.05 ± 0.52	20.51 ± 0.37	23.58 ± 0.35 ***	20.13 ± 0.31	20.69 ± 0.78
Linoleic acid (C18:2n6)	13.83 ± 1.54	13.36 ± 1.44	14.80 ± 0.72	14.08 ± 1.85	13.18 ± 1.39
GLA (C18:3n6)	1.85 ± 3.31	1.01 ± 1.31	1.59 ± 1.45	0.57 ± 0.47	1.47 ± 1.26
DGLA (C20:3n6)	2.06 ± 0.23	1.94 ± 0.27	1.48 ± 0.09 *	1.87 ± 0.20	1.91 ± 0.01
AA (C20:4n6)	11.01 ± 0.24	10.89 ± 1.26	8.977 ± 0.16 **	10.66 ± 0.24	11.27 ± 0.30
EPA (C20:5n3)	0.91 ± 0.07	0.96 ± 0.13	0.74 ± 0.04 *	0.99 ± 0.14	0.99 ± 0.03
DHA (C22:6n3)	7.66 ± 0.88	8.53 ± 0.51	6.73 ± 0.21 ***	7.36 ± 0.01 ***	8.85 ± 0.08
Total PUFAs	39.09 ± 3.53	38.56 ± 1.24	36.28 ± 1.64	38.34 ± 0.16	39.44 ± 0.27
**Index**					
PI	126.8 ± 1.54	135.3 ± 1.95 ###	112.5 ± 0.78 ***	125.9 ± 1.57 ***	133.1 ± 0.87
SCD1	0.86 ± 0.06	0.93 ± 0.01	0.91 ± 0.01	0.80 ± 0.02 *	1.00 ± 0.08

Table 1 Data presented as mean ± standard deviation (SD). Statistical analyses: one-way ANOVA Dunnet’s post hoc analysis: TGF-β co-treated HE compared to TGF-β treated slices (* *p* < 0.05; ** *p* < 0.001; *** *p* < 0.0001; ### *p* < 0.0001). Abb: SFAs: Saturated Fatty Acid; MUFAs: Monounsaturated Fatty Acid; GLA: γ-linolenic acid; DGLA: Dihomo γ-linoleic acid; AA: Arachidonic acid; EPA: Eicosapentaenoic acid; DHA: Docosahexaenoic acid; PUFAs: Polyunsaturated Fatty Acid; PI: Peroxidation index; SCD1: Stearoyl-CoA Desaturase-1.

**Table 2:** Unique Assay ID of primers used to measure the expression genes of interest.

Gene	Unique Assay ID	Chromosome Location	Amplicon Length
*α-SMA*	qMmuCID0006375	19:34246173-34248479	178
*COL1A1*	qMmuCID0021007	11:94936401-94937999	104
*SERPINH-1*	qMmuCID0022896	7:99347103-99348831	153
*Fibronectin-1*	qMmuCED0045687	1:71597314-71597404	61
*IL-1β*	qMmuCID0005641	2:129366041-129367295	95
*IL-6*	qMmuCID0005613	5:30018087-30019454	112
*β-ACTIN*	qMmuCED0027505	5:142904472-142904610	109

## Data Availability

Data are available from the corresponding author upon reasonable request.

## Supplementary Materials

The following supporting information can be downloaded at: https://www.mdpi.com/article/10.3390/ijms27020594/s1.

## Abbreviations

ECM	Extracellular Matrix
HCC	Hepatocellular Carcinoma
HSCs	Hepatic stellate cells
α-SMA	Alpha Smooth Muscle Actin
TNF-α	Tumor Necrosis Factor alpha
IL-1β	Interleukin-1 beta
IL-6	Interleukin-6
TGF-β1	Transforming Growth Factor beta 1
pSMAD2	Phosphorylated SMAD2
SMAD2	SMAD Family Member 2
H&E	Hematoxylin and eosin
SI	Silver Impregnation
PCLSs	Precision-Cut Liver Slices
ROS	Reactive Oxygen Species
Hepa-RG	Human Hepatoma Cell Line
COL1A1	Collagen Type I Alpha 1 Chain
COL1A2	Collagen Type I Alpha 2 Chain
RT-qPCR	Reverse Transcription quantitative Polymerase Chain Reaction
FN-1	Fibronectin-1
SERPINH-1	Serpin Family H Member 1
VIM	Vimentin
PI	Peroxidation index
PUFAs	Polyunsaturated Fatty Acids
MUFAs	Monounsaturated Fatty Acids
DGLA	Dihomo γ-linoleic acid
AA	Arachidonic Acid
EPA	Eicosapentaenoic Acid
DHA	Docosahexaenoic Acid
GLA	Gamma-linolenic Acid
SCD1	Stearoyl-CoA Desaturase-1
FBS	Fetal Bovine Serum
DMSO	Dimethyl Sulfoxide
HBSS	Hank’s Balanced Salt Solution
β-Actin	Beta-actin
RIPA buffer	RadioImmunoPrecipitation Assay buffer
CCl4	Carbon Tetrachloride
BDL	Bile Duct Ligation
MMP.2	Matrix Metalloproteinase 2
MMP.9	Matrix Metalloproteinase 9
TIMP-1	Tissue Inhibitor of Metalloproteinase 1
TIMP-2	Tissue Inhibitor of Metalloproteinase 2
NPAs	NAFLD Promoting Agents
NAFLD	Non-Alcoholic Fatty Liver Disease
NASH	Non-Alcoholic Steatohepatitis
