# Supplementary material for: Anti-Fibrotic and Anti-Inflammatory Effects of Hesperidin in an Ex Vivo Mouse Model of Early-Onset Liver Fibrosis
Source: Int J Mol Sci. 2026 Jan 7;27(2):594. doi: 10.3390/ijms27020594 (PMC12840767; doi:10.3390/ijms27020594)
Supplement: Supplementary file 1 [file ijms-27-00594-s001.zip › ijms-3995574-supplementary.pdf]

Supplementary Materials

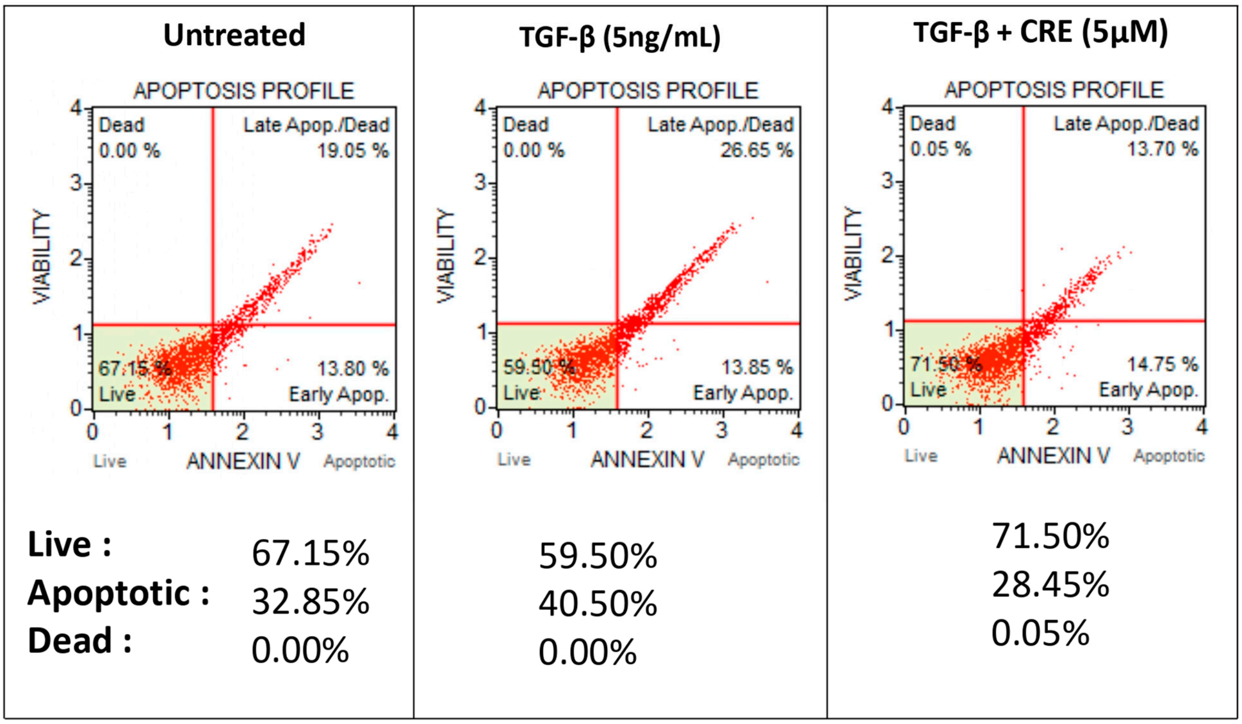

Figure S1. Representative dot plots of flow cytometry analyses are shown for the untreated condition (Untreated), TGF-β treatment (5 ng/mL, 48 h), and Crenigagestat (5 µM), used as a positive control, to contextualize the efficacy of HE within the experimental system.

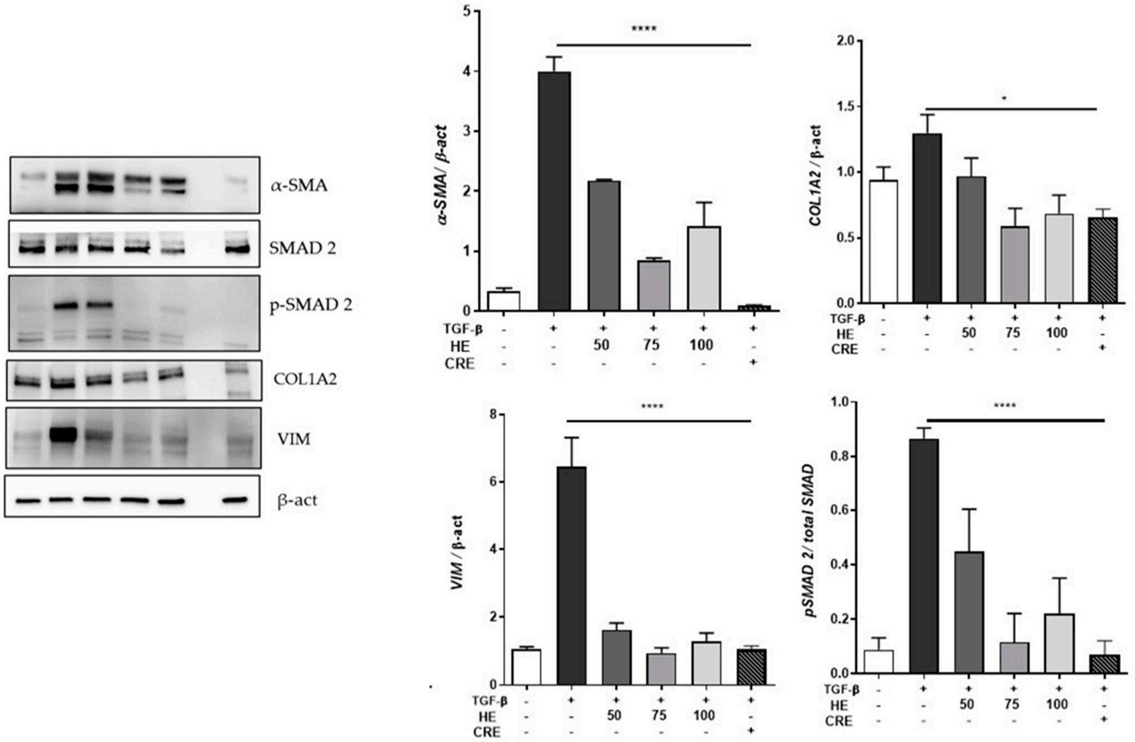

Figure S2. The images show the protein expression of α-SMA, COL1A2, and VIM, as well as the p-SMAD2/SMAD2 ratio, in PLSCs treated with TGF-β (5 ng/mL, 48 h) in the absence or presence of Crenigacestat (CRE, 5 μM), used as a positive control. As expected, CRE treatment significantly reduced the protein expression of all the fibrotic markers analyzed. All data reported in each panel are presented as mean ± SEM from three independent experiments (n = 3 for each condition). Statistical analysis: one-way ANOVA with Dunnett's post-hoc test: PLSCs co-treated with CRE compared to PLSCs treated with TGF-β (* $p < 0.05$, **** $p < 0.0001$).

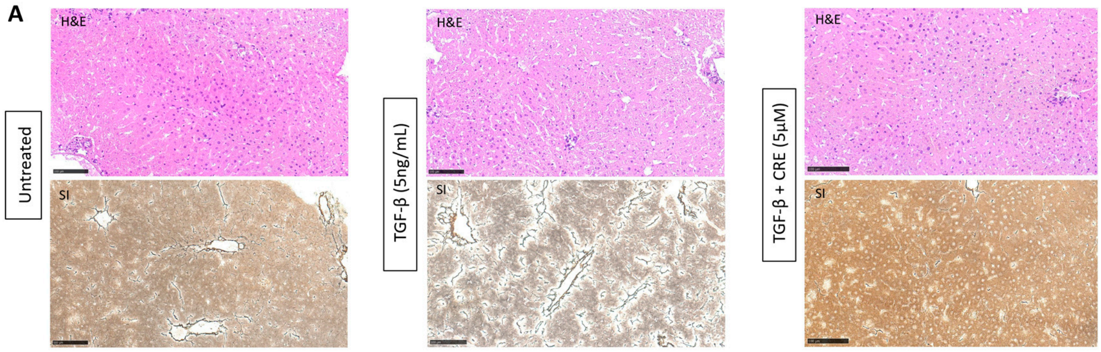

Figure S3. H&E staining and reticulin-silver impregnation (SI) were performed on hepatic sections after 48 h of treatment with TGF-β1 (5 ng/mL) and co-treatment with CRE (5 μM). As shown in the figure, the marked increase in reticular fiber infiltration in the hepatic parenchyma induced by TGF-β1 was reduced in the presence of CRE, suggesting an attenuating effect of the drug on reticulin remodeling.
